# Systems Human Immunology and AI: Immune Setpoint and Immune Health

**DOI:** 10.1146/annurev-immunol-090122-042631

**Published:** 2025-04

**Authors:** Yona Lei, John S. Tsang

**Affiliations:** 1 Yale Center for Systems and Engineering Immunology and Department of Immunobiology, Yale University School of Medicine, New Haven, Connecticut, USA; 2 Department of Biomedical Engineering, Yale University, New Haven, Connecticut, USA; 3 Chan Zuckerberg Biohub NY, New Haven, Connecticut, USA

**Keywords:** systems immunology, immune variation, immune setpoint, machine learning, artificial intelligence, AI, immune health, human immunology

## Abstract

The immune system, critical for human health and implicated in many diseases, defends against pathogens, monitors physiological stress, and maintains tissue and organismal homeostasis. It exhibits substantial variability both within and across individuals and populations. Recent technological and conceptual progress in systems human immunology has provided predictive insights that link personal immune states to intervention responses and disease susceptibilities. Artificial intelligence (AI), particularly machine learning (ML), has emerged as a powerful tool for analyzing complex immune data sets, revealing hidden patterns across biological scales, and enabling predictive models for individualistic immune responses and potentially personalized interventions. This review highlights recent advances in deciphering human immune variation and predicting outcomes, particularly through the concepts of immune setpoint, immune health, and use of the immune system as a window for measuring health. We also provide a brief history of AI; review ML modeling approaches, including their applications in systems human immunology; and explore the potential of AI to develop predictive models and personal immune state embeddings to detect early signs of disease, forecast responses to interventions, and guide personalized health strategies.

## 1 INTRODUCTION

The immune system plays critical roles in the health and function of all organs throughout the life span; its dysfunction has been implicated in most if not all human diseases. In addition to defending against pathogens and tumors, the immune system acts as a sensor of physiologic stress, monitoring and integrating both internal and environmental signals to mount appropriate responses to maintain homeostasis (1–3). The immune system thus provides an informative window into an individual’s health and physiologic state.

Technological advances over the past two decades, including single-cell multiomics, proteomics, and immune cell phenotyping, have opened new avenues for interrogating the immune system at unprecedented levels of depth and resolution (4). When applied to the most readily accessible human tissues, such as blood, skin, and airway, these technologies provide a wealth of information about an individual’s immune state in health and disease (1). Variability is a fundamental hallmark of human immunity. Diverse factors sculpt each individual’s unique immunological states (5), driving variable responses to pathogens, vaccines, and treatments to result in a wide spectrum of health outcomes. This heterogeneity can pose challenges; for instance, vaccines may not be universally effective (6). However, population variation is essential to empower the development of predictive models of immune system behavior (5, 7), which can enable the identification of intervention targets (7, 8). Thus, understanding and harnessing human immune variation are central to human immunology and are crucial for developing and optimizing personalized interventions and outcomes.

**Figure 1:**
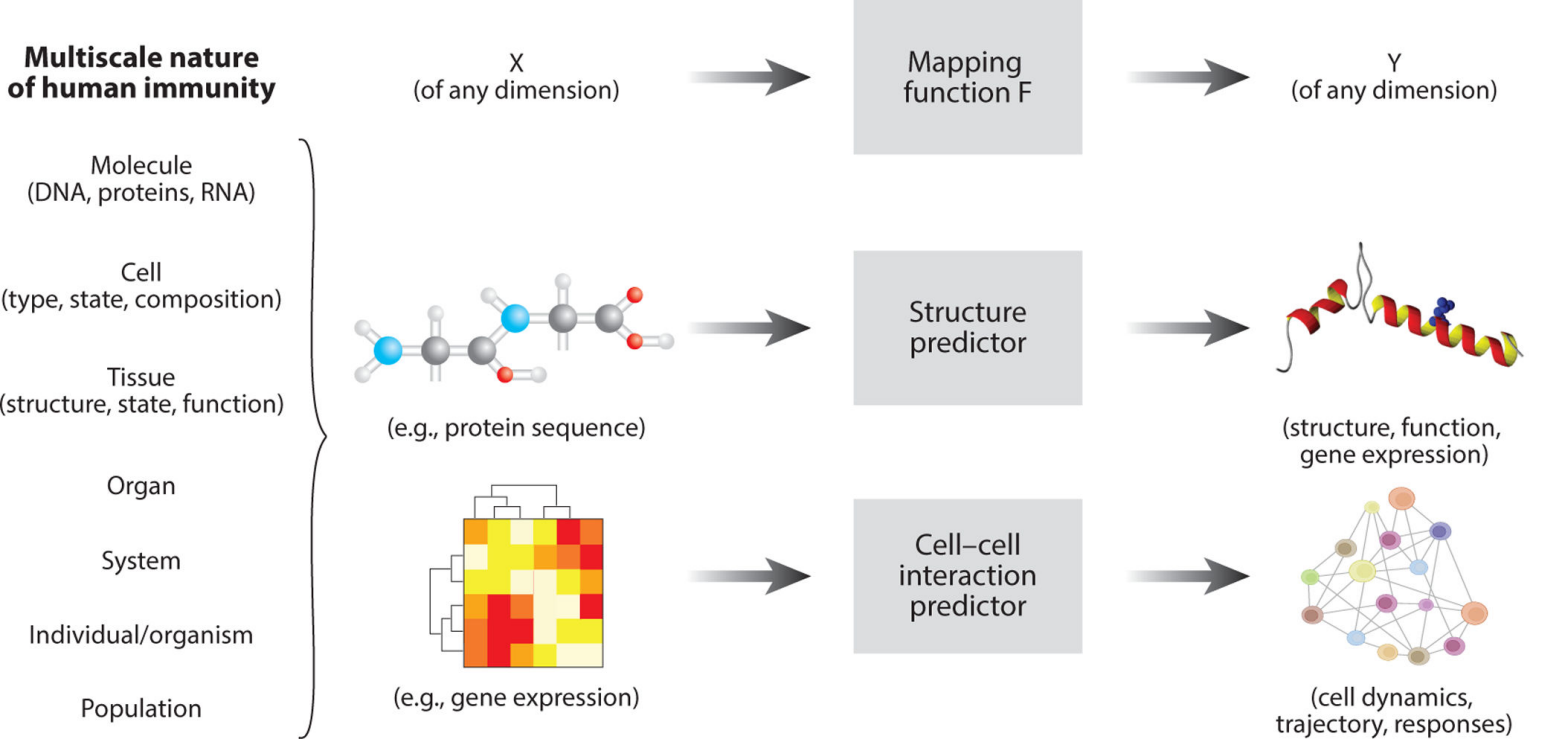
Artificial intelligence (AI) framework for decoding multiscale immune system complexity. Given any input data, machine learning (ML) algorithms can automatically learn a mapping function that transforms input X of any dimension into output Y of any dimension. ML offers a unifying framework for uncovering and mapping complex input–output relationships in the immune system. The human immune system orchestrates defenses and maintains homeostasis across multiple levels of biological organization, ranging from molecular and cellular interactions to tissue and organismal responses and, ultimately, to population-level phenomena. Information processed at one level of the immune system can be integrated to shape responses in and across other levels and bodily systems. ML approaches have been developed to map input–output relationships and extract biological insights both within and across these scales. For example, at the molecular level, ML algorithms have been developed to map protein sequence (input) to three-dimensional structure (output), assess variant effects on protein function, and predict gene expression patterns from sequence data. More generally, such an ML framework can be employed to effectively map various input features to functions, including the mapping of gene expression programs to cell types, dynamics, and functional responses at the cellular level, as well as the mapping of personal immune states to functional responses at the organismal level. Thus, AI/ML-driven approaches facilitate the integration and analysis of diverse data types, enabling predictions of immune outcomes across different scales. Such a comprehensive framework fosters a more holistic, predictive, and mechanistic understanding of human immunity and holds the potential to advance precision medicine.

Modern artificial intelligence (AI) and particularly machine learning (ML) have recently emerged as powerful tools for deciphering complex biological systems (9, 10). As high-throughput technologies continue to yield a deluge of immunological data across biological scales, ML approaches offer the capability to extract biologically meaningful relationships, reduce data dimensions, build predictive models, and define biomarkers and potential mechanistic determinants of immune statuses and health outcomes (11). The ability of ML models to compress high-dimensional data such as those capturing immune system states (with many configurations) into smaller representations (or embeddings) is key to deciphering and harnessing human immune variation and building powerful predictive models. Not only can these compressed representations enhance predictive capacity, but also they can facilitate the discovery and mechanistic modeling of biological players and patterns not immediately apparent in higher dimensions (11, 12). Thus, AI may provide both a unifying conceptual paradigm—decoding molecular and cellular logic within and across biological scales (Figure 1)—and powerful tools to enable systems human immunology, whose mission is not merely to transform ever-larger data sets into biological insights and provide tools for precision medicine but also to achieve a quantitative, mechanistic understanding of the human immune system together with its interaction with physiology as a holistic system.

In this review, we explore the landscape of systems human immunology and AI, focusing on recent advances in investigating human immune variation and the concepts of immune setpoints and immune health, which are critical anchors linking personal immune states and functional outcomes. We then provide a brief history of AI/ML and an overview of predictive modeling approaches. Finally, we offer our perspective on how emerging technologies and AI may drive progress toward the systems human immunology vision, including the exciting prospect of leveraging the immune system as a sensor of health by developing predictive models of immune setpoints and immune health. These models could detect signs of disease long before symptom onset (13–15), predict responses to interventions (7, 16, 17), and chart future health trajectories. Such predictive capacity could empower precise interventions to prevent diseases, enhance responses, and guide lifestyle choices.

## 2. HUMAN IMMUNE VARIATION

Human immunity exhibits extensive variability, manifesting both across individuals and within a single person over time (5, 18). Examples and consequences of public health importance abound, including experimental malaria vaccines that showed high protective efficacy when evaluated in the United States but whose efficacy dropped significantly when tested in Africa (19). Reduction in immunogenicity has also been reported for other vaccines, including bacille Calmette-Guérin (BCG) (20, 21) and yellow fever (22), although these observations do not suggest that individuals in Africa have less robust immune responses; for example, Africa as a continent appeared to have fared better in COVID-19 outcomes in comparison to the United States and Europe (23). While these differences could be attributed to many factors, variation in individual immune statuses and responses plays a crucial role. More broadly, variability is pervasive in diverse contexts, including responses to cancer immunotherapy (24, 25) and other therapeutics (26, 27), as well as the magnitude and frequency of disease activities (or flares) observed in autoimmune conditions such as lupus (28, 29).

### 2.1. Intrinsic Variation and Heritability

**Figure 2:**
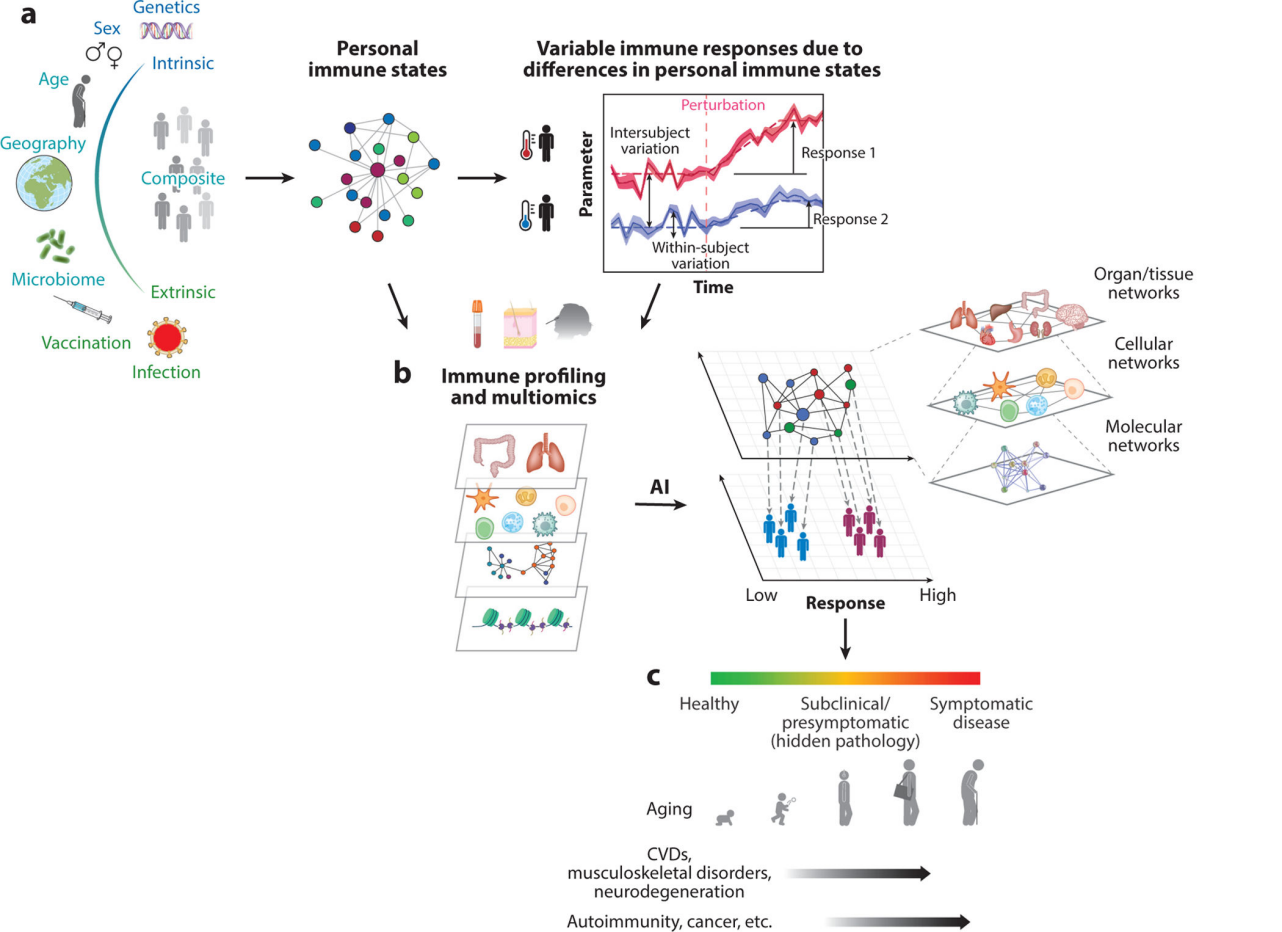
Integrating multimodal immune profiling and artificial intelligence (AI) to investigate human immune variation and predict immune responses and immune health. (*a*) Personal immune states, or setpoints, are dynamically shaped by intrinsic (e.g., genetics, sex), extrinsic (e.g., microbiome, vaccination, infection), and composite (e.g., age, geographical location) factors, which often reflect contributions from multiple sources (e.g., age reflects development, senescence, and exposure). Given differences in these diverse factors, immune states and responses vary widely across individuals. (*b*) Immune states can be assessed through multimodal profiling of accessible clinical samples from sources such as blood, skin, and nasopharyngeal tissues. The resulting large, high-dimensional data capture the statuses of immune components and networks spanning tissue, cellular, and molecular levels. Given the large number of variables (e.g., more than a trillion immune cells in the human body; 93) and the interactions among them, the number of configurations (or immune states) is vast. AI, including deep learning, can decipher the complex correlation structure of such data and compress this vast configuration space into lower-dimensional embeddings that capture essential relationships among key immune entities. Such representations of personal immune states will not only elucidate the potential mechanistic drivers of immune variation but also enhance our ability to make accurate, personalized predictions of immune responses to perturbations—such as vaccination, infection, disease, and therapeutics—as well as health trajectories. (*c*) Predictors or setpoints of immune responses can serve as a proxy for measurements of immune system health, enabling us to chart individuals along a spectrum of immune health (*color bar*). As shown recently (15), such immune health metrics could track healthy aging and capture subtle signals (e.g., deviation from healthy homeostasis) to report on “hidden” pathology in clinically healthy individuals before overt symptoms manifest. Such early detection is particularly critical for combating chronic diseases, which often progress silently for years. The use of the immune system as a sensor for identifying physiological deviations and disease processes in their incipient and early stages (progression is shown by the thick arrows, going from healthy to less healthy to overtly pathological) opens a window for early intervention that could alter disease trajectories and improve long-term health outcomes. Abbreviation: CVD, cardiovascular disease.

Immune variation is driven by a complex interplay among heritable and nonheritable factors, which together shape molecular, cellular, and tissue states in an individual to influence physiological functions and responses (18, 30) (Figure 2a). Genome-wide association and twin studies have revealed genetic drivers of immune parameters such as circulating immune cell frequencies and their disease associations (31, 32). The extent of genetic contribution depends on the immune parameter and type of response. Twin studies suggest that antibody responses to early childhood vaccines are more heritable than those in adults (33, 34); presumably, as genetically identical twins age, their immune systems diverge due to nonheritable contributions from cumulative environmental exposures. Indeed, immune cell frequencies, circulating cytokine levels, and antibody responses to seasonal influenza vaccination are largely nonheritable in adults (35), although certain cell phenotypes seem highly heritable (36). Certain changes in these immune parameters likely involve the epigenome, which exhibits plasticity in response to environmental stimuli. Epigenetic modifications can alter gene expression programs in immune cells, thus influencing cellular phenotypes and the regulatory networks that regulate immune responses. In general, it can be challenging to ascertain whether epigenetic mechanisms are causal for or merely correlated with responses, although another twin study showed that 70% of the chromatin modification variations can be explained by nonheritable factors (37), suggesting that environmental exposures together with other variables (e.g., sex) can mediate their effects on immune functions through epigenetic mechanisms. Thus, nonheritable factors are key determinants of a person’s immune state and function.

Variables intrinsic to a person, such as sex, can shape immune states and functions. Sex differences have been observed in susceptibility to infection and autoimmune disease, vaccination responses, and treatment outcomes, which could be consequences of sex dimorphic innate and adaptive immune parameters and responses (38, 39). For example, females tend to have elevated early inflammatory responses and subsequently increased immunogenicity to vaccination (39). At the cellular level, the frequency and activity of immune cells can differ between the sexes. Males typically have higher natural killer cell frequencies (40), whereas females tend to have more B cells that perhaps explain their greater antibody responses to vaccination regardless of age (38, 40). Men with elevated testosterone levels exhibit an immunosuppressive transcriptional signature enriched for genes involved in lipid biosynthesis and have blunted influenza vaccine responses (41). The health consequences of these sex differences, including increased prevalence of autoimmunity and organ rejection posttransplantation in females, can be profound (38).

### 2.2. Development, Aging, and Cumulative Exposures

The immune system undergoes extensive development and environment-driven transformation throughout an individual’s life span (42, 43). Thus, many immune parameters and functions are linked to age. The immune system of infants and children is different from that of adults, although much remains to be learned regarding immune variation within and across individuals as a function of age. Early life exposures sensed by the immune system are thought to be key determinants of health trajectory later in life, including susceptibility to immune-mediated diseases and infections (43). Interestingly, a longitudinal, system-level analysis of peripheral blood mononuclear cells from preterm and term human newborns revealed that, despite large initial differences, their immune states converged toward a shared developmental trajectory at 3 months of age (44). However, this robust, stereotypical immune system development during early life can be hampered by gut bacterial dysbiosis, highlighting the importance of the microbiome in shaping the development of the immune system and immune variation. The observation that the newborn immune state varies widely but could converge later in time suggests that the immune system is a versatile sensory system that can buffer variability in inputs to robustly generate the desired output, potentially through feedback mechanisms. A genetically hardwired system would presumably be evolutionarily less advantageous because it would be less adaptable to diverse immunological challenges. However, given the large number of immune parameters and the much larger combinatorial space of immune states, this early convergence in the frequency and phenotype of certain peripheral immune cell populations does not imply that variation across individuals is minimized. We have yet to fully unravel the complexities of pediatric immune development and how individuality is established, maintained, and shaped over time. Indeed, poorly understood phenomena important for public health, such as early-life imprinting of antigen-specific immunity (45, 46), variable durability of childhood vaccine–induced immunity (47), and antigen-agnostic effects of live vaccines such as BCG (48, 49), underscore the value of achieving a quantitative and predictive understanding of early life and pediatric immune variation.

After reproductive age, a process often called immunosenescence drives immune aging and the related concept of inflammaging (50–53). Immunosenescence is characterized by systemic chronic, low-grade inflammation (51, 53), impaired T cell activation and metabolism (54), decreased B cell frequencies, and defects in class-switch recombination and somatic hypermutation (55). These features are linked to the decreased magnitude and quality of antibody responses in the elderly (56, 57), increased infection susceptibility and mortality, and heightened risk of diseases such as cancer (58–60). In a comprehensive longitudinal immune profiling of older versus younger adults, Alpert et al. (61) showed that not only do older individuals have distinct and more rapid dynamical changes as they age but also their immune state predicts the future rate of change of certain immune aging variables. These analyses thus revealed an aging trajectory that allowed an immune age metric to be derived, and this metric could predict all-cause mortality in an independent cohort. The concept of an immune aging metric (62) is reminiscent of the desire to pinpoint an individual’s “biological” instead of chronological age to assess health and predict future trajectory (63–67). In fact, our recent multimodal analyses of 22 monogenic immune-mediated diseases (15) suggest that the immune changes associated with healthy aging are concordant with those observed in diverse human pathologies. Thus, deviations from health in diverse conditions, including aging, have shared systemic immune consequences (see Section 3.3, below).

The impact of age on immunity further emphasizes that life histories and environmental exposures (or variables extrinsic to the individual), including infections, the microbiome, and variables associated with geographic location, cause immune variation. Systems immune profiling of 670 healthy individuals aged 2 to 86 years identified cohabitation as the largest contributor of interindividual variation in immune cell composition, with 50% less variation between individuals with a shared environment (68). This study found that sex-based differences at the molecular level were more pronounced than those at the cellular level (68, 69), suggesting a complex interplay whereby variation in molecular parameters can be buffered by compensatory or feedback mechanisms that result in lower variability at the cellular level, although the functional implications are not yet clear.

Chronic or latent infection status can also modulate immune states and responses (5). For instance, cytomegalovirus seropositivity is associated with enhanced antibody responses to influenza vaccination in young, healthy individuals, but it has no effect on older individuals who exhibit decreased responses independent of their cytomegalovirus status (70). The role of the microbiome has also come to light (71, 72). A trial administered broad-spectrum antibiotics to healthy adults followed by seasonal influenza vaccination and systems immunology analyses (73). Surprisingly, despite extensive microbiome loss, these individuals appeared capable of mounting normal responses without diminishing antibody outputs. Mildly impaired antibody responses were observed in individuals with low baseline levels of neutralizing antibodies. Furthermore, antibiotic treatment resulted in enhanced proinflammatory signatures in circulating immune cells before vaccination, resembling the early transcriptional responses to influenza vaccination observed earlier in the elderly (73, 74). While it remains to be determined whether preexisting antibodies are causal, these observations highlight the potential vulnerability of populations with lower preexisting immunity, including infants and older adults, to mount inadequate vaccine responses after microbiome perturbation.

### 2.3. Geography as a Composite Variable

Geographic location is a complex, composite variable in that it reflects contributions from myriad factors including ancestry/genetics, diet, and pathogen load (75). To date, systems human immunology studies investigating immune variation have been conducted largely in adults from a few high-income countries such as the United States. Thus, their findings may not generalize to other regions, a limitation that is particularly relevant given that infectious diseases disproportionately affect individuals in low- and middle-income countries (76). To address this issue, Hill et al. (77) conducted longitudinal immune phenotyping of peripheral blood mononuclear cells from children participating in a large malaria vaccine (RTS,S) trial conducted in Tanzania and Mozambique. A comparison between African and Dutch children revealed that while certain immune cells follow a similar developmental trajectory, the dynamic changes in immune cell frequency can vary between countries, even those in close geographic proximity, such as Tanzania and Mozambique. Tanzanian children had fewer circulating T follicular helper cells and plasmablasts at baseline, which were linked to lower IgG titers following RTS,S vaccination. Anemia emerged as a potential driver of these immune differences, with higher prevalence in Tanzanian children. Anemia was associated with reduced B cell responses, including lower levels of IgG memory B cells and plasmablasts following vaccination, implying that iron deficiency negatively affects vaccine responsiveness (77). One limitation of this study is that both the sample and data of the Dutch cohort were generated independently of the RTS,S malaria vaccine trial in Africa, making it difficult to attribute the observed differences solely to geographic variation. More broadly, the RTS,S vaccine trial was conducted in seven African countries, and vaccine efficacy varied across study sites (78–80). Similarly, attenuated live malaria sporozoite vaccines have variable efficacy across populations, particularly between Africa (low protection) and the United States/Germany (high protection) (19, 81, 82), underscoring the importance of understanding human immune variation across populations to pursue goals such as reversing vaccine hyporesponsiveness (75). Much larger cohorts spanning variables such as geographic region, age, and sex, together with coordinated human immune profiling and AI model building, are needed to unravel the determinants and circuitry underlying human immune variation around the globe (83).

## 3. IMMUNE SETPOINT AND IMMUNE HEALTH

Immune variations span different types and manifest at diverse spatial and timescales. As discussed above, together they can be conceptualized as intrinsic (e.g., genetics, sex), extrinsic (e.g., exposure, season), or composite variables that together shape personal immune states (e.g., molecular, cellular, and tissue statuses and their interactions), which in turn determine physiologic and functional outputs (e.g., response to vaccination, infection, disease) (Figure 2a). Linking personal immune states to functional outputs is the concept of immune setpoints, which correspond to immune states of an individual that determine (and thus predict) specific functional output quality and quantity. The setpoint concept originated from control theory, where a setpoint is a variable whose target (or homeostatic) value is maintained by a control system. An example is the set ambient temperature of a room regulated by an air-conditioning system. A setpoint can be multidimensional (e.g., temperature, pressure), and as exemplified by temperature, the setpoint shapes the environment in which activities and processes occur. As defined, different individuals in the human population can have different setpoint states at a given point in time, thus providing different molecular, cellular, and tissue environments within individuals in which responses are orchestrated to generate functional outputs (e.g., antibody and cytotoxic T cell responses to challenges) (5). A personal immune setpoint can be continuously shaped by genetic, environmental, and physiological signals (5, 28, 84–86).

The complexity of the individual immune setpoint is further shaped by the remarkable diversity of T and B cell receptor (TCR and BCR) repertoires. Monozygotic twins, despite sharing identical genetics, exhibit distinct antigen receptor repertoires due to the stochastic nature of V(D)J recombination and differences in antigen-driven selection processes (87). This receptor diversity contributes significantly to variations in immune responses between individuals, including differential responses not only to vaccines and pathogens but potentially to other diseases such as autoimmunity and neurodegeneration (88–90). The repertoire continues to evolve throughout life, influenced by aging and cumulative antigen exposure, further modulating the immune setpoint (91, 92). Thus, the theoretical configuration space of human immune states is vast, given that there are approximately two trillion immune cells in the human body (93) and each cell can exist in diverse functional states, including variable antigen recognition receptors in B and T lymphocytes—not to mention interactions among these cells and with other cell types throughout the body (Figure 2a,b). However, the actual state space is likely much smaller due to functional and evolutionary constraints, and the setpoint state space should have even lower dimensionality given that the variables that shape and predict specific responses are even more constrained.

### 3.1. Poised and Natural Adjuvanted Setpoint

Baseline setpoint predictors have been reported for vaccination responses (7, 28, 94–98), therapy outcome (24, 99), and disease activity or progression (13, 28). An emerging concept in the context of vaccine responses is that baseline setpoints can resemble innate immune signatures induced by vaccine adjuvants. Individuals possessing such “natural adjuvanted” baseline setpoints tend to have more robust responses to vaccination. Our recent multimodal single-cell analysis of two influenza vaccines—one formulated with the adjuvant AS03 and one without an adjuvant (85)—demonstrated this concept, revealing that high responders to the unadjuvanted vaccine exhibit baseline states mirroring those specifically elicited by AS03 1 day after vaccination in myeloid dendritic cells and CD14^+^ monocytes. Further investigation found that CD14^+^ monocytes from high responders at baseline displayed elevated phosphosignaling responses to in vitro lipopolysaccharide (LPS) stimulation, reflecting an increased cell-intrinsic response capacity. Together, these observations suggest that the naturally adjuvanted setpoint may drive more robust vaccine responses by enhancing innate immune cell sensing capacity. While other baseline predictors of vaccine responses have not been compared explicitly with early immune responses elicited by adjuvants, these baseline setpoints also tend to mark inflammation and innate immune cell activation, suggesting that a more “poised” immune state is a common feature of baseline setpoints. However, the details can differ, and the underlying mechanism likely involves not only a single cell type or cell-intrinsic process but also a network of interacting cell states. For example, we found a baseline setpoint involving activation of plasmacytoid dendritic cells as well as both B and T lymphocytes; this baseline setpoint predicted not only antibody responses to influenza and yellow fever vaccination but also plasma cell–associated flares in lupus (28). Despite the low abundance of plasmacytoid dendritic cells in blood, this setpoint can be measured via as few as 10 blood transcripts thanks to the correlated cell states: Signals from the more abundant B and T lymphocytes in blood, including but not limited to their interferon response gene signatures, are sufficient to capture the state of the correlated cell network. This observation also suggests that setpoints for autoimmunity (encompassing disease status, activity, endotype) and other immune response outcomes, including those to infection and vaccination, could be shared (100, 101). Understanding what perturbations could change and modulate these setpoints to influence disease risk and activity could help unravel the association between infection and autoimmunity (e.g., Epstein–Barr virus and multiple sclerosis; 102) and identify drug targets to mitigate autoimmune disease activity. Uncovering natural adjuvanted setpoints may also guide the development of vaccine adjuvants.

### 3.2. Setting the Setpoint

Analyses of blood from healthy individuals over weeks to years (68, 95, 103) suggest that immune cell frequencies and serum protein levels, including baseline setpoints predictive of influenza vaccination (95), are relatively stable, although these variables can exhibit temporal trends during pediatric development or aging later in life (42, 61, 104, 105). In general, aside from antigen-specific or bystander memory lymphocytes, most if not all of the acute challenge–induced immune changes are expected to revert to their prechallenge baseline state (18), a process governed by tightly regulated cellular circuitry tailored to each person’s unique physiological context. The degree of temporal stability varies across immune parameters, with some components uniquely shaped by prior immunological challenges or exhibiting more pronounced fluctuations than others. Furthermore, immune setpoints can be modulated. BCG vaccination and fungal infections can change chromatin states in monocytes and myeloid progenitor cells to confer antigen-nonspecific effects on future responses, a phenomenon known as trained innate immunity (48). The induced nonspecific, protective effects are thought to occur through modification of epigenetic states in progenitor cells, including those reflected by histone marks such as H3K4me1, H3K4me3, and H3K27ac (48, 106). Severe COVID-19 can induce similar long-term changes to circulating monocytes and hematopoietic stem and progenitor cells (HSPCs), including increased activities in transcription factors like interferon regulatory factors, hyperresponsiveness to inflammatory stimulations, metabolic reprogramming, and increased inflammatory cytokine production (107, 108). Since circulating monocytes have average half-lives of only a few days (109), longer-term memory can reside in the HSPCs that give rise to monocytes (110). The wider impacts of such long-term alterations of HSPCs—such as whether and how they could limit the production of normal, nontrained monocytes and other HSPC progeny beyond monocytes—remain to be determined.

Longitudinal multiomics analyses of healthy individuals who recovered earlier from mild COVID-19 and were not hospitalized revealed that male recoverees exhibited a more poised baseline immune state associated with stronger innate and adaptive responses to subsequent influenza vaccination (84). This male-specific setpoint state involved multiple interacting immune cell populations, including virtual memory-like CD8^+^ T cells and classical monocytes, that form a mutually reinforcing positive feedback circuit via IFN-γ and IL-15. This finding suggests that prior viral infections could establish new, sex-dependent setpoints with the potential to shape future responses in an antigen-agnostic manner. Similarly, analyses of human responses to H5N1 vaccination with or without the AS03 adjuvant illustrated that even an antigen-sparing, unadjuvanted vaccine alone can shift the immune system to a new baseline setpoint associated with enhanced responses (111). Upon AS03-adjuvanted H5N1 influenza vaccination, classical monocytes can also attain a refractory state associated with a global reduction in histone acetylation and chromatin accessibility, which persisted for at least a month after vaccination. These monocytes had a heightened antiviral program and were more resistant to viral infections (112). These studies suggest that infection, vaccination, or any immunological challenge can alter immune setpoints, thereby potentially modulating future responses. Along with better knowledge of the causal mechanisms underlying baseline immune setpoints, these studies could reveal actionable intervention targets, thus raising the prospect of not only predicting outcomes but also modulating the baseline setpoint to enhance subsequent immune responses (7). As revealed by a recent drug screen in mouse bone marrow–derived macrophages, even noninflammatory (classically thought of as anti-inflammatory) agents such as glucocorticoids can induce trained immunity phenotypes (113). Other promising immune setpoint modulators include drugs targeting epigenetic regulators (e.g., histone deacetylase inhibitors), which have shown potential in treating hematologic and solid tumors (114) and altering innate immune training (115), as well as microbiota-targeted strategies that could promote the efficacy of immune checkpoint inhibitors (116, 117).

### 3.3. Setpoint and Immune Health: Probing Hidden Health Deviations

The concept of an immune setpoint is closely related to the idea of personal immune health. For example, predictors of responsiveness to vaccines and therapeutics, as well as resistance to infections and severe disease, naturally give rise to intuitive measures of immune health and competence (Figure 2c). More broadly, defining and measuring immunological health are still in their infancy. One approach is the concept of immunological resilience based on age-independent adaptation to antigenic stimulation; in other words, immune systems that are best able to return to homeostasis after antigenic stimulation such as infection are the most immunologically healthy (118). When challenged with inflammatory stressors, individuals who resist the degradation of immunological resilience tend to have lower risks of disease acquisition. Another approach is to define immunological health by learning common features of diverse pathologies. We pursued this idea by integrating multiomics and ML to simultaneously assess 22 monogenic immune-mediated diseases (15). Despite the penetrance and relatively large effect of monogenic mutations, interindividual variation in diverse immunological parameters remains the dominant type of variation, significantly above those attributable to age, disease condition, and medication use. Another unexpected observation was that unsupervised ML—not knowing who has what disease or any other phenotypic attributes, but solely utilizing individual-level multiomics data—generated a one-dimensional metric capable of distinguishing between sick patients and age- and sex-matched healthy controls. This same metric emerged with the use of data from sick patients alone (or from healthy controls only, albeit less robustly), suggesting that it captures a robust and potentially meaningful measure of immune states. Indeed, this immune health metric (IHM) was concordant with the probability of being healthy derived using supervised ML distinguishing disease from health. In independent data sets, the IHM marks healthy aging; measures biological age declines; forecasts age-dependent decreases in vaccination responses; and predicts deviations from health, including autoimmunity, heart failure, and poor body mass index. Given that immune cells surveil the body and can detect signals of stress and deviation from physiologic homeostasis in tissues, the IHM could perhaps capture such signals to report on “hidden” pathology. Thus, as we observed earlier (15), even clinically healthy individuals can be distributed over a wide range along the immune health axis, from very healthy to healthy, to inching toward pathology but still without overt signs of disease, and finally to having overt disease (Figure 2c). Many diseases, including Alzheimer’s and autoimmunity, as well as aging-associated declines, take years to develop into full-blown symptomatic conditions (119–124). Further development of the IHM concept and the identification of additional dimensions of immune health could enable early detection and intervention to revert early health deviation and functional dysregulation.

### 3.4. Tissue Setpoint

Thus far, our discussion of human immune variability and setpoints has focused on blood-based assessments reflective of peripheral (or systemic) immune states. The nearly two trillion immune cells in the average human body reside and function largely in tissues, particularly the bone marrow, the lymphatic system, and barrier sites (93, 125, 126). Thus, understanding tissue setpoints and deciphering the connection between blood and tissue immune states are major research goals. Certain immune cell populations are rarely found in blood under normal physiological conditions; these include tissue-resident macrophages that develop directly from embryonic progenitor cells and certain memory T cells that establish long-term residency in tissues. The extent to which tissue immune states can be captured in blood by cells, proteins, metabolites, and so forth is an active area of investigation. For instance, some circulating protein components of the IHM might have originated from tissues. However, many tissue-specific responses remain difficult to detect, as circulating immune parameters often poorly reflect the immune activity present in tissues. For example, longitudinal immune profiling of paired blood and airway samples from patients with severe COVID-19 showed that T cell frequencies in the airway, but not the blood, predict outcomes in patients with severe COVID-19 (127).

Tissue setpoints are determined by the intrinsic properties and states of tissue components, which determine how tissues respond to perturbations. While variable outcomes of identical perturbations are commonly observed, we are only beginning to understand how preexisting tissue states influence these responses. Studies of mucosal immune responses, such as those of the upper airway, have provided illuminating examples. When clinically healthy, nonsmoking adults were experimentally challenged with respiratory syncytial virus (RSV), inflammatory activation of neutrophils in the nasal mucosa before virus exposure predicted symptomatic RSV disease (128). This baseline setpoint state was associated with the suppression of early mucosal immune responses, particularly IL-17 and related pathways, during the early presymptomatic phase of the response. In contrast, individuals who resisted infection exhibited presymptomatic activation of IL-17- and tumor necrosis factor–related pathways and were able to mount an early IL-17-driven inflammatory response that might have controlled and blocked viral infection. Along similar conceptual lines, another study showed that children had higher basal levels of innate immune genes, including viral sensors, in upper airway epithelial cells, macrophages, and dendritic cells (129). This finding may explain why young children are often better protected against SARS-CoV-2 symptomatic infection: Presumably they are better primed for virus sensing, leading to stronger innate activation upon infection (129).

The broader tissue environment can be shaped by previous experiences or exposures, creating a kind of tissue memory that influences future responses. For example, after acute skin inflammation, epithelial stem cells retain inflammatory memory that accelerates barrier restoration during subsequent tissue damage (130). This memory is mechanistically maintained through sustained changes in chromatin accessibility at key stress response genes activated by the initial stimulus. Genes governed by these persisting accessible regions are transcribed rapidly in response to secondary stressors. While these alterations mobilize stem cells more rapidly and are often associated with accelerated wound repair, they are also associated with increased susceptibility to autoimmune disorders and cancer. The concept of tissue memory has also been extended to nonimmune cell progenitors in other tissues, including intestine, respiratory tract, and pancreas, with implications for infection, tumorigenesis, and chronic inflammatory diseases (131–134).

Similar to immune setpoints in blood, tissue setpoints can be shaped by intrinsic, extrinsic, and composite factors and underlie interindividual variation in response to immune perturbations. For example, individuals with chronic respiratory and allergic diseases are at increased risk of severe outcomes from respiratory viral infections. One study demonstrated that nasal challenge with a Toll-like receptor 7/8 agonist led to heightened interferon and chemokine responses in nasal mucosa in individuals with allergic rhinitis (with or without asthma) compared with healthy participants (135). This finding suggests that baseline inflammation and immune dysregulation shape tissue-specific immune responses and thereby influence the susceptibility to and outcome of respiratory viral infections.

## 4. PROBING HUMAN IMMUNE SYSTEM STATES

### 4.1. Perturb, Then Measure

Natural immune variation, together with perturbations such as vaccination followed by high-dimensional immune profiling and statistical and ML analyses, has been key to empowering the discovery of predictive immune setpoints in humans (1, 5, 7). In addition to being an effective medical intervention, vaccines can serve as a perturbation to probe the human immune system, allowing assessment of whether baseline immune variation across individuals can lead to differences in functional outputs (Figure 2a). This approach has uncovered setpoints that predict and potentially determine outcomes (7). Vaccines naturally elicit in vivo responses linked to function, including early innate and adaptive responses (e.g., days 1, 3, and 7 following influenza vaccination), as well as longer-term impacts on the immune system, such as the magnitude and durability of antibody, memory T cell, and trained innate immunity responses (5, 48). Vaccines have also been used to probe patients to assess how disease-associated immune changes may influence responses; such in vivo perturbations, together with in vitro cellular stimulation assays (see Section 4.3), can reveal molecular and cellular defects that become apparent only when evaluating responses to perturbations (84, 85, 136–138). Therapeutics such as checkpoint blockades, cytokines, and cellular therapies are additional perturbations that can be applied in appropriate populations to identify immune setpoints.

### 4.2. Time: A Critical Dimension

As mentioned above, the immune system is inherently dynamic, and immune parameters can change over time at various scales. These changes range from highly stable parameters (i.e., those whose temporal variations are significantly less than differences across individuals) to transient changes to long-lasting shifts, potentially influencing future immune responses or having no discernible impact—effects that are often difficult for cross-sectional snapshots alone to reveal. Longitudinal studies with thoughtful study designs are thus desirable for capturing the temporal dimension of immune states and identifying robust predictors and setpoints of functionally relevant outcomes. A key design decision is the temporal sampling frequency, which is often constrained by budget and logistics. By taking repeated measurements over time, one can observe how each person’s immune parameters evolve with or without perturbation, allowing for the identification of temporally stable versus varying traits that may be population or individual specific, as well as temporal changes that could be the key drivers of immune variation observed in cross-sectional studies. Denser sampling over hours to days can help unveil response dynamics including specific molecular, pathway, and cellular parameters, as highlighted by early response dynamics to vaccination (111) and neonatal development during the first week of life (139). Sampling in the range of weeks to months can provide both innate and adaptive response dynamics after a perturbation (5, 140) and reveal temporally stable parameters when there are no apparent perturbations. For example, setpoint parameters predictive of vaccine responses revealed by our earlier research tended to be temporally stable within healthy individuals for more than 2 months (28, 95). Unexpectedly, longitudinal sampling of patients with different monogenic diseases (with samples collected months to 2 years apart) suggested that many blood parameters—including transcriptional profiles, cell frequencies, and circulating protein levels—are stable, varying significantly less over time within a person compared with the extent of differences observed across individuals. Variations attributable to individuals are also substantially higher than those explained by monogenic disease labels, suggesting that individual immune state is the dominant unit of variation.

The study of development, aging, seasonality, and so forth naturally requires sampling at timescales ranging from months to years. In addition, the temporal sequence captured in longitudinal data provides an opportunity to disentangle causal relationships and infer the directionality of effects (8, 141, 142). Strategic cohort design can address specific facets of immune system behavior; for example, household and twin studies can help distinguish environmental and genetic effects on immune states and functions (31, 35, 36, 68, 143, 144). However, large cohorts encompassing various demographics are essential in order to capture and utilize a broad spectrum of setpoints and influencing factors. These cohorts not only ensure proper representation of under-represented populations but also leverage natural population variation to increase the power for novel discoveries (5, 7, 18).

### 4.3. Sample Type, Multimodality, and Single-Cell Analysis

Modern multiomics and immune-profiling technologies allow comprehensive measurements of human immune system states and behavior (Figure 2b). Together, these approaches can perform measurements at different biological scales, from the genome and epigenome to cells, tissues, and physiologic phenotypes. Blood is the most common sample type from which circulating molecules such as cytokines and immune cells can be interrogated, and similar data can be obtained from other body fluids, including saliva and urine. While integrative analyses of blood and tissue samples remain sparse at the cohort level, they could provide novel insights into cell trafficking and the extent of mirroring of immune statuses between blood and tissues (145–147). Biopsies from accessible tissues such as skin and gut are becoming more common (125, 148–150), but longitudinal sampling of children and clinically healthy individuals remains more challenging. More specialized samples such as draining lymph node aspirates or biopsies (151–155) enable the evaluation of germinal center reactions and other processes in secondary lymphoid organs; swabs from mucosal sites such as the nasopharynx have proven informative for dissecting viral infection responses and protective neutrophil and memory T cell setpoints (128, 156, 157). Recent data suggest that cell-free DNA/RNA from blood could report on tissue status (158, 159); in the future, advances in cellular recording technologies could enable remote sensing of tissue status in blood and other body fluids (160–162).

While sample type and amount can limit downstream measurements, modern multiplexed technologies require relatively small inputs to generate large data sets, as exemplified by targeted proteomic technologies like SomaScan (163–165) and Olink (166, 167). Advances in mass spectrometry and enrichment approaches have also enabled both targeted and nontargeted profiling of proteins and metabolites (1, 168–171). The measurement of cells is a pillar of immune profiling. It can involve assessing the frequency and composition of immune cells as well as their molecular profiles, activation and proliferation status, effector functions, antigen specificities, and cytokine production. The surge of single-cell omics has enhanced our ability to resolve cell type–specific behaviors and states with remarkable granularity and resolution. These methods generate extensive quantities of multimodal data to interrogate multiple aspects of cellular immunity.

Single-cell multiomics technologies are continually evolving to increase both throughput and modalities that can be profiled in a single experiment, enabling the simultaneous profiling of the transcriptome, proteome, epigenome, metabolome, and so on (4). A notable example is cellular indexing of transcriptomes and epitopes by sequencing (CITE-seq), which combines multiplexed surface protein detection via nucleic acid–based barcodes with transcriptome profiling (172) and, optionally, V(D)J sequencing to simultaneously capture the variable receptor and antibody repertoires in T and B cells. Surface protein expression profiles allow the use of decades of immunological knowledge and data to annotate immune cells and states pioneered by flow cytometry (4, 173, 174). CITE-seq has also been extended to the detection of specific TCRs and BCRs via barcoded antigens (175, 176) and to the labeling of intracellular proteins and their posttranslational modifications to assess signaling status and transcription factor activities (177). In principle, there is no limit to the number of targets that can be detected, but both cost and the availability of off-the-shelf barcoded antibody reagents are practical constraints. Additional modalities, including chromatin accessibility, have been added to CITE-seq or single-cell RNA sequencing to enhance the multimodal characterization of cells (178, 179).

While costs have come down dramatically, they continue to be a major limitation in the application of single-cell multiomics to longitudinal human cohort studies. Thus, the number of cells that can be profiled per sample is still relatively low compared with that of technologies such as modern high-dimensional mass or flow cytometry, which remain staples of the human immune-profiling toolkit as they can measure up to ∼50 parameters in tens of thousands of cells per sample with high throughput (180, 181). Moreover, mapping of the interactions between single cells within tissues offers insights into cell–cell interactions and the role of the microenvironment in shaping cellular behavior. Single-cell omics methods typically require the dissociation of cells from their native tissue, resulting in the loss of spatial information. Spatial technologies bridge this gap by enabling the simultaneous profiling of multiple modalities down to the single-cell level while maintaining the spatial coordinates of cells within intact tissues (4, 182). While multimodal experimental methods such as CITE-seq enable direct linking of multiple modalities to the same individual cells, numerous single-cell data sets are unpaired, with different modalities profiled from cells of the same sample or tissue but not from the exact same single cells (183). Emerging tools are beginning to address data set heterogeneity and reconcile the differences between omics-specific feature spaces (173, 184).

In addition to analyses of cells and molecules, in vitro stimulation of cryopreserved immune cells (185–187) or whole-blood cultures (188, 189) followed by signaling and transcriptional response measurements provides another layer of information. Stimulations include cytokines such as interferons, TCR signaling activation triggers (e.g., anti-CD3/CD28), and ligands for pattern recognition receptors such as LPS. When single-cell omics or flow/mass cytometry is used, responses can be measured at different timescales and down to single-cell resolution. Signaling responses such as phosphorylation states are typically measured at early time points to measure cell-intrinsic responsiveness, which can reveal baseline immune setpoints associated with aging (103), pediatric development (190), and vaccine responses (85). Transcriptional responses measured at later time points can be more challenging to interpret, given the influence of other cells in the culture and upstream responses in the same cell. Secreted molecules such as cytokines can also be interrogated by analyzing cell culture supernatant. As discussed above with regard to naturally adjuvanted baseline setpoints (85), comparison of in vitro stimulation responses of cells from the baseline with in vivo innate responses elicited by vaccination can lead to biological insights.

## 5. AI AND SYSTEMS HUMAN IMMUNOLOGY

The sheer volume, high dimensionality, and multimodality of immune-profiling data (e.g., capturing variations across individuals over time with high molecular and cellular resolution) require advanced AI/ML approaches to extract biological insights (10, 12).

### 5.1. A Brief History of AI

While the field of AI has no definitive beginning, one early milestone was in 1950, when computing pioneer Alan Turing proposed a test to evaluate whether a computer exhibits behavior indistinguishable from that of humans (191). John McCarthy, a mathematician and another AI pioneer, coined the term AI in 1955, when he was preparing for the first conference in AI (192). For many years, AI focused on knowledge representation and ways for computers to mimic how humans think, resulting in probabilistic and logic-based representations of the world and associated algorithms (193). Another branch of AI, often called pattern recognition, focused on approaches such as neural networks (NNs) and learning algorithms, including classic statistical methods like linear regression and decision trees (see Section 5.2). This branch evolved into what is now called ML (Figure 1), which aims to learn mathematical functions that map a vector of numbers (inputs) into another vector of numbers (output).

### 5.2. A Brief Introduction to Machine Learning

**Figure 3:**
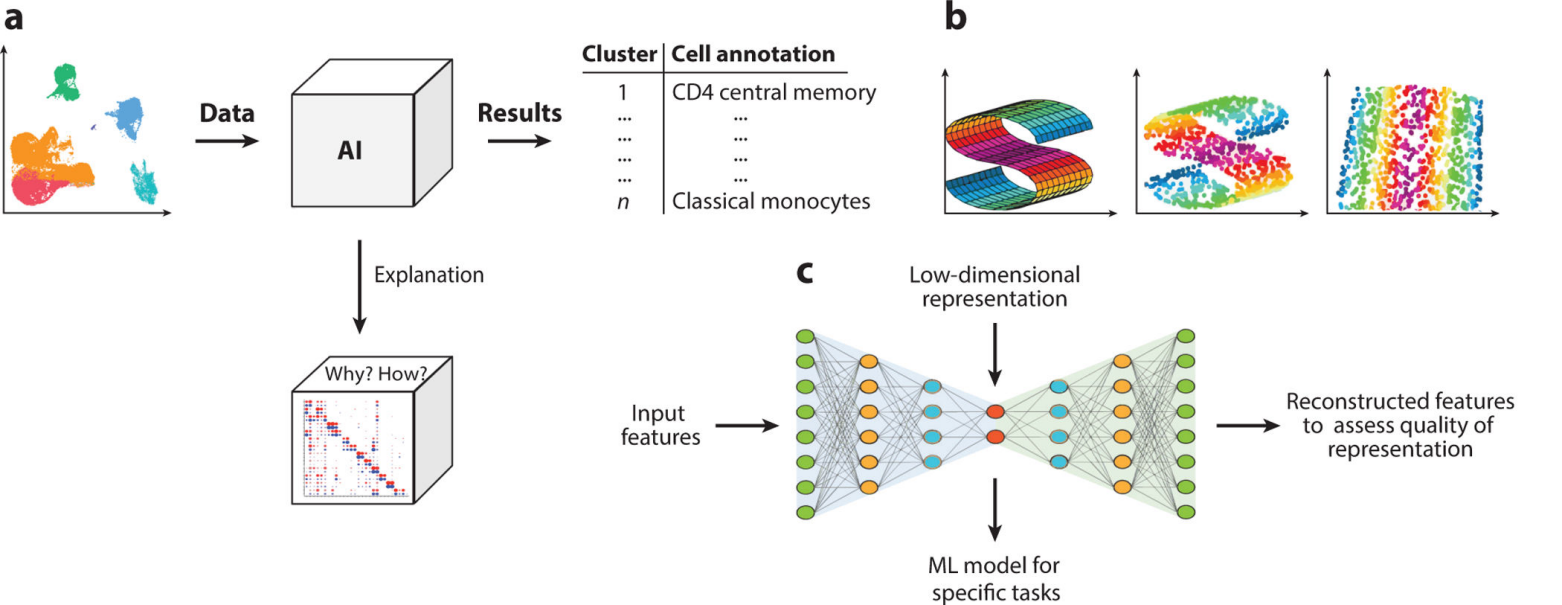
Interpretable machine learning (ML) approaches for high-dimensional immune data analysis. (*a*) ML models can be classified into explainable versus black-box models. ML models are trained to map input data to output; the reasoning and path to reaching decisions or computing the output remain largely hidden in black-box models but can be fully or partly retrieved in explainable or interpretable models, such as random forest classification or regression models. In the context of cell type prediction using single-cell transcriptomic data, a black-box model might accurately predict that a cell belongs to a specific immune cell type or subset on the basis of its gene or protein expression patterns, but without revealing which specific expression patterns inform this classification. Such a lack of interpretability makes it challenging both to verify whether the model’s decision-making is consistent with biological knowledge and to extract potential new knowledge and insight from the model. In contrast, explainable AI models provide interpretable insights, so when applied to predicting cell types and states, such models allow users to examine the relative importance and contribution of different features or combinations of features in determining each cell type assignment. (*b*) The “curse of dimensionality” refers to the challenges that arise in analyses of high-dimensional data, such as immunological measurements where each sample is characterized by tens of thousands of features. As the dimensionality increases, data points become extremely sparsely distributed, making it extremely challenging to identify meaningful patterns or relationships (in ML lingo: Learning mapping functions that can interpolate the data values in the vast space without any observed data is a challenging task). However, many features measured at higher dimensions may be redundant (e.g., correlated with other features) or irrelevant for a given predictive task, thus adding noise that can obscure the true biological signals. Dimensionality reduction techniques, or representation learning, address these challenges by mapping high-dimensional data into a lower-dimensional space while revealing the underlying manifold structure using fewer features. These learned representations transform the original feature space into new informative features, enabling more informative visualizations. Importantly, these new features serve as inputs for downstream learning tasks that can benefit from the compressed and more informative representations (or embeddings). In this hypothetical example, a representation learning procedure can compress and “unfold” the data in three-dimensional space into a new representation in two dimensions. The new representation captures the topological structure of the data, allowing easy classification of the data points belonging to different color categories (essentially, a linear, cutoff-based function will successfully delineate the points). Such a classification function would be significantly more challenging to learn in the original three-dimensional space. (*c*) While traditional dimensionality reduction techniques, such as principal component analysis, are useful for transforming data into lower dimensions, they are often limited to capturing linear relationships (i.e., summing up contributions from features) and may not fully capture complex nonlinear relationships or topological features. Neural network models, such as autoencoders, can overcome these limitations by learning nonlinear mapping functions that compress high-dimensional input features into low-dimensional representations that preserve biologically meaningful information. In this case, the preservation of information is formally trained and assessed via a decoder, which aims to reconstruct the original data from the low-dimensional representation. The extent to which the original data can be reconstructed from the compressed representation also provides a way to assess the quality of the learned representations, ensuring their biological relevance. The low-dimensional representations can be used for downstream ML tasks, such as clustering, classification, or prediction of outcomes. Panel *b* adapted from Reference 194 (CC BY 4.0). Panel *c* adapted from Reference 195.

Uncovering immune setpoints and potential determinants of functional outcomes can be framed as an ML/predictive modeling problem, in which the inputs are the data on molecular and cellular immune states of a person and the goal is to develop ML models that use the input data to predict individual outcomes (Figure 1). For example, given the transcriptional state of T cell subsets in blood before anti-PD-1 treatment, a model could predict whether the patient will respond to the treatment. ML models can be divided into interpretable versus less interpretable or black-box models; the main difference is that the key parameters contributing to the prediction can readily be extracted in the former, while it is more challenging to do so in the latter (Figure 3a).

Examples of interpretable models include linear regression and random forest models; in general, NN and deep neural network (DNN) models are less interpretable. An NN is a class of functions that mimics how the brain computes. The simplest unit, called a perceptron, multiplies each input value by a weight (which is learned from the training data), sums up these weighted values, and computes the output by basically evaluating whether the weighted sum is above or below a certain threshold (196). While there are variations in how individual units compute and many different architectures are possible, an NN essentially comprises many perceptron units where the output of one unit feeds into the input of other units. Units are often organized in layers, and a DNN contains many layers; learning in such deep networks is computationally demanding. However, such networks are remarkably expressive (or capable), allowing them to learn to perform extremely complex data transformations, such as translating text between two natural languages.

NN-based approaches have revolutionized biology in recent years, yielding breakthroughs in protein structure prediction (197), artificial protein design (198), and prediction of pathologic genetic mutations (199, 200) (Figure 1). Thanks to the abundance of single-cell data, DNN models of cells including immune cells are emerging (201, 202), even though their predictive capacity and generalizability are still being evaluated (203). In general, given the complexity of the human immune system and the variability in the human population, a hurdle AI still faces is the relative dearth of data covering the vast space of personal immune and molecular recognition states in individuals and diverse populations.

In addition to interpretability, ML algorithms and associated models can be classified as either supervised or unsupervised (204). In supervised learning, the algorithm is trained on labeled data, where inputs are paired with their ground-truth outputs. It learns to map the input features to the correct output labels, allowing the model to make predictions on new data based on the learned patterns. A key goal is to achieve generalizability, which refers to the ability of the resulting model to predict the output with good accuracy and precision when given unseen input (12). By contrast, in unsupervised learning, the model learns patterns and structures from unlabeled data and is typically used for clustering, dimensionality reduction, and anomaly detection.

Unsupervised learning can serve as a first step toward the discovery of structure or patterns in the data, which can then be combined with the original labeled data set to enhance the performance of the subsequent supervised ML step. This process is closely related to a key ML approach called representation learning (205), which aims to overcome the “curse of dimensionality,” a problem that arises when working with high-dimensional immune state measurements (Figure 3b). ML models are inherently data interpolation schemes in that they attempt to learn mathematical functions to fill in the unseen data points (or data volumes) in the input data space. A single-variable input is easy to interpolate even after seeing a relatively small quantity of training data—when a linear model is used, this corresponds to the classic single-variable regression problem. However, the data volume grows exponentially as a function of the number of input dimensions. Thus, given the large number of immune-profiling parameters that are now routinely being measured in systems human immunology studies (minimally in the 10^5^–10^6^ range when transcriptomics data are used), interpolation becomes extremely challenging. Representation learning aims to discover compressions of high-dimensional data into lower-dimensional spaces to capture the correlated structure and relationship between the input features. The resulting, more succinct representations, or embeddings, are often more useful encodings of the input data, such that downstream supervised learning becomes easier and more robust (Figure 3c). This paradigm has been successfully applied in classical ML and statistical modeling, such as principal component analysis followed by linear regression. In modern ML, it is exemplified by methods such as deep autoencoders (206), which first derive latent representations of the input data and then feed these representations into additional NN layers to perform supervised learning and downstream predictive tasks (207).

### 5.3. Methods Linking Personal Immune States to Functions and Outcomes

Supplemental Table 1 lists the key ML techniques used in systems human immunology studies to uncover predictive insights on human immune setpoints and immune response predictors. Classic regression-based approaches are often used to model relationships between input and output variables and to quantify the input variables’ effects on functional outputs of the immune system. These approaches include linear regression for continuous outcomes (72, 208), logistic regression for binary outcomes (28, 209), and generalized linear models to incorporate diverse distributions beyond the normal (84, 210). Regression-based models can also be extended to handle more complex data structures and relationships. For example, mixed effects models, which incorporate both fixed and random effects—the former can model contributions from variables such as sex, while the latter can account for effects such as stable baseline differences across individuals—are useful for analyzing hierarchical data structures and repeated measures common in immunological studies, such as longitudinal vaccine responses (85, 211) and clinical outcomes (212). We recently used this approach to model the hierarchical variations arising from multimodal single-cell data obtained from different individuals sampled longitudinally over time before and after vaccination (85). Moreover, regularized regression methods such as lasso and ridge regression are well-suited to handling multicollinearity, or correlations between predictors. They reduce model complexity and prevent overfitting by applying penalties to regression coefficients, making them particularly valuable for analyzing the many correlated variables generated by immune profiling, including single-cell multiomics (204).

Although ML tools excel at extracting potentially meaningful patterns that might be obscured by the messiness of biological systems, they cannot inherently prove causal relationships. While one could drop out specific input variables (or combinations thereof) to computationally assess their contributions to prediction performance, rigorous experimental validations are required to elucidate the mechanistic basis, establish causality, and provide feedback to refine and improve computational models. The synergy between computational and experimental methods fosters an iterative cycle positioned to catalyze deeper insights and drive the field toward more accurate predictions and clinically transformative applications.

## 6. PERSPECTIVE AND CONCLUDING REMARKS

The integration of systems human immunology and AI is poised for significant advances, which will build on current methodological, technological, and conceptual developments to improve our understanding of human immune variation. As we look ahead, several emerging trends will shape the advancement of the field. Addressing the historical bias in systems immunology studies toward relatively small cohorts in economically more advanced regions, large-scale studies are slowly expanding the scope to include traditionally underrepresented populations and a broader range of variables shaping human immunity (83), including factors related to urban and rural environments (75). To enhance data comparability and reproducibility across diverse research sites and populations, initiatives are underway to develop globally applicable and replicable standardized immune-profiling kits and protocols (83, 213–215; see https://www.humanimmunomeproject.org/scientific-approach). These efforts could lead to a unified framework for immune profiling, spanning sample collection, processing, and data reporting. Such standardization will not only facilitate seamless data integration and reduce cost but also enhance the robustness and generalizability of immunological findings. Adding to highly standardized immune profiling, in vivo immune cell–based sensors represent an exciting frontier (216; see https://www.czbiohub.org). Immune cells such as T cells surveil diverse tissues and are thus natural sensors of tissue and physiologic states. Harnessing such microscale biosensors both naturally and synthetically via engineered immune cells could lead to real-time, high-resolution monitoring and reporting of physiologic changes within the human body (217). These technologies could lead to the generation of massive temporal and spatial data sets within and across individuals, which would fuel advanced computational and AI modeling, potentially revealing new immune health dimensions to enable early disease detection and precise immune modulation strategies.

### 6.1. Toward Human Immune State Embeddings

While current statistical and ML methods have been instrumental in identifying predictors of immune features and outcomes, as the multiplicity and complexity of data modalities increase together with more extensive longitudinal profiling of ever larger populations, the complex, nonlinear relationships inherent in the resulting high-dimensional data sets will demand more expressive ML models, such as DNNs. DNN models are adept at scaling effectively and often show more pronounced performance gains with increasing data size, leveraging their deep architectures to learn richer, more nuanced representations. Building on these strengths, foundation models—DNNs with a very large number of parameters trained in an unsupervised fashion on massive, diverse data sets—that have been developed for genomes (218), proteins (219, 220), and cells (201–203, 221, 222) could become a core tool to transform heterogeneous data into predictive immunological insights. They also facilitate the exchange of knowledge and AI models across immunological contexts through transfer learning (223). The few-shot learning capabilities (i.e., requiring very few additional training examples for new applications) and support for fine-tuning of foundational models enable quick adaptation to new immunological subdomains and predictive tasks, even when data specific to that subdomain are limited (224). We envision that DNNs and other emerging AI advances will provide novel representations or embeddings of human immune states and their correlation structure to empower a predictive and, ultimately, causal mechanistic understanding of the human immune system and its interactions with physiology. Achieving such informative, compressed representations also has practical implications: The number and type of immune state measurements could be dramatically reduced by focusing on these key embedding states. Doing so would lead to highly standardized immune profiling with reduced cost and enhanced robustness and could thus allow for a significant expansion of cohort sizes and sampling frequency.

### 6.2. Toward Effective Integration of Data-Driven and Mechanistic Models

Successful integration of AI in systems immunology is not without challenges. As discussed above, ML models such as DNNs often function as black boxes, making their predictive and decision-making processes inscrutable (225). The lack of interpretability hinders our ability to extract causal, mechanistic insights that can guide further hypothesis generation and experimentation. Without a clear understanding of how the model arrives at its predictions, identifying and mitigating inherent biases become exceedingly difficult. When models generate incorrect predictions or exhibit unexpected behavior, interpretability is essential for pinpointing the root cause and driving model refinement. These issues collectively contribute to limited trust and adoption among researchers and clinicians, who justifiably expect concrete proof that ML predictions are grounded in biologically relevant features and relationships (226). Moreover, most ML models are designed to recognize patterns and correlations in the data but lack the inherent ability to discern whether these relationships are causal (12, 227). For example, correlations between cytokine levels and observed immune responses do not necessarily imply causation. Such findings, though valuable for prediction, require further investigation to establish causal relationships in immune system behavior. These limitations underscore the need to integrate complementary approaches such as quantitative mechanistic models, which extend beyond statistical associations to simulate and interrogate immunological processes bottom up (228, 229). These models are typically constructed on the basis of existing knowledge of the central players in a particular process or, more rarely, by incorporating key molecular and cellular components identified through statistical and ML-based approaches. These models can be formulated, parameterized, and simulated computationally using agent-based approaches, systems of ordinary (or partial) differential equations (230), or the Gillespie algorithm (231), for example. Thus, mechanistic models can in principle predict how molecular-level changes can propagate across scales over time under a variety of conditions, which would be impractical to test experimentally (229). However, a major challenge is the large number of parameters with unknown values; how they vary across individuals in the population is also largely unknown. New concepts are needed to bridge this scale gap: Often, top-down, data-driven approaches powered by AI result in features that are not easy to model directly via mechanistic modeling approaches. Perhaps AI will again come to the rescue; for example, by relatively sparse sampling of the parameter space of stochastic mechanistic models, as discussed above, ML can learn the mapping between parameter configuration and phenotypic outputs of the system under simulation (232, 233). Thus, we envision that, to bridge this top-down (AI- and data-driven) versus bottom-up (mechanistic modeling) scale gap, ML can map human immune state embeddings to molecular and cellular state embeddings to reveal key dynamic variables for incorporation into mechanistic models.

Systems human immunology has made remarkable strides in delineating human immunological diversity and health. The convergence of high-throughput technologies generating massive data and AI is setting the stage for mapping and predicting the dynamic landscape and trajectory of individual human immunity and physiologic health. Envisioning the path ahead, interpretable AI models capable of forecasting immune responses based on an array of individual factors—including immune profiles, ancestry, age, sex, socioeconomic factors, and other pertinent information represented as personal immune state embeddings—hold immense promise. Emerging research strategies and tools could propel us toward a future where immune health is precisely monitored, predicted, and modulated.

## Supplementary Material

supplementary material
