# Supplementary material for: Systems Human Immunology and AI: Immune Setpoint and Immune Health
Source: Annu Rev Immunol. Author manuscript; Available in PMC 2026 Jul 18. (PMC13380186; doi:10.1146/annurev-immunol-090122-042631)
Supplement: supplementary material [file NIHMS2167445-supplement-supplementary_material.pdf]

Supplemental Material: Annu. Rev. Immunol. 2025. 43: https://doi.org/10.1146/annurev-immunol-090122-042631

Downloaded from www.annualreviews.org. Yale University (ar-150330) IP: 130.132.173.234 On: Tue, 07 Jul 2026 16:58:55

Supplemental Table 1. Statistical and machine learning (ML)-based techniques with examples in immune response prediction

Method	Type	Application	Examples
Linear regression	Supervised, interpretable	Prediction of continuous outcomes.	Predict antibody titer after vaccination (1).<br>Assess relationships between disease activity and microbial dysbiosis (2).
Logistic regression	Supervised, interpretable	Prediction of binary outcomes.	Predict immune protection from infection (3).<br>Predict immunotherapy response (4, 5).<br>Predict high vs. low responders to vaccination (6).
Partial least squares regression	Supervised, interpretable	Prediction with many correlated predictors, maximizing covariance between predictors and outcomes.	Identify features most predictive of disease severity/outcome (7).
Generalized linear models	Supervised, interpretable	Modeling outcomes with flexibility for various data distribution types.	Identify baseline signatures predictive of post-vaccination interferon responses (8).
Mixed effect models	Supervised, interpretable	Analysis of grouped or hierarchical data incorporating both fixed and random effects, including designs with multiple samples from the same individuals.	Model vaccine responses across individuals while adjusting for other factors (9).<br>Identify signatures associated with COVID-19 severity and mortality (10).
Lasso regression	Supervised, interpretable	Regularization for feature selection by shrinking some coefficients to zero.	Identify features distinguishing long COVID patients from others (11).

Downloaded from www.annualreviews.org. Yale University (ar-150330) IP: 130.132.173.234 On: Tue, 07 Jul 2026 16:58:55

Ridge regression	Supervised, interpretable	Regularization for multicollinearity, reducing overfitting by shrinking coefficients of correlated predictors.	Predict chronic kidney disease severity (12). Predict overall survival and treatment response in ovarian cancer patients (13).
Principal component analysis (PCA)	Unsupervised, interpretable	Linear dimension reduction, identifying major variation patterns.	Reduce complexity of antibody repertoires and cluster patients by disease states or immune responses (14).
t-distributed stochastic neighbor embedding (t-SNE)	Unsupervised, less interpretable	Non-linear dimensionality reduction, preserving local structure.	Visualize high-dimensional data in low-dimensional space (15).
Uniform manifold approximation and projection (UMAP)	Unsupervised, less interpretable, belonging to the same class of models as t-SNE.	Non-linear dimensionality reduction, preserving local and global structure.	Visualize high-dimensional data in low-dimensional space (16).
Joint and individual variation explained	Unsupervised, interpretable	Dimensionality reduction and integrated analysis of multi-omics data, quantitating joint (shared across data modalities) and individual modality variations.	Distinguish between clinically healthy individuals and patients (17).
Hierarchical clustering	Unsupervised, interpretable	Clustering, revealing hierarchical relationships between features or samples.	Identify immune cell subtypes associated with breast cancer survival outcomes (18).
k-means clustering	Unsupervised, interpretable	Partitioning data into distinct clusters based on distance to the nearest cluster center.	Identify tumor subtypes used to predict immunotherapy efficacy (19).
Support vector machine	Supervised, less interpretable	Classification and regression by finding optimal boundaries that separate data into distinct categories.	Predict cancer survival and adjuvant benefit (20).

Random forest	Supervised, interpretable	Ensemble classification, regression, and feature importance ranking.	Establish a metric that can distinguish healthy individuals and patients (17). Predict vaccine response based on pre-vaccination signatures (21).
Gradient boosting	Supervised, interpretable	Ensemble classification and regression, sequentially building simple models and iteratively improving on errors.	Predict COVID-19 disease severity (22).
k-nearest neighbors	Supervised, interpretable	Classification and regression based on similarity to nearby data points.	Predict lupus disease activity (23).
Deep learning models	Supervised or unsupervised, less interpretable	Complex pattern recognition in high-dimensional data, automated feature extraction, classification, regression.	Predict cancer monotherapy or drug combination therapies (24). Predict polypharmacy side effects (25).
Autoencoders	Unsupervised or semi-supervised, less interpretable	Dimensionality reduction, feature learning, data denoising, anomaly detection.	Predict chemotherapy response (26). Microbiome-driven prediction of immune checkpoint inhibitor responsiveness (27).2/20/2025 8:58:00 AM

References

1. Matsuura T, Fukushima W, Nakagama Y, Kido Y, Kase T, et al. 2024. Factors impacting antibody kinetics, including fever and vaccination intervals, in SARS-CoV-2-naïve adults receiving the first four mRNA COVID-19 vaccine doses. *Sci Rep*. 14(1):7217

Downloaded from www.annualreviews.org. Yale University (ar-150330) IP: 130.132.173.234 On: Tue, 07 Jul 2026 16:58:55

Downloaded from www.annualreviews.org. Yale University (ar-150330) IP: 130.132.173.234 On: Tue, 07 Jul 2026 16:58:55

2. IBDMDB Investigators, Lloyd-Price J, Arze C, Ananthakrishnan AN, Schirmer M, et al. 2019. Multi-omics of the gut microbial ecosystem in inflammatory bowel diseases. *Nature*. 569(7758):655–62

3. Khoury DS, Cromer D, Reynaldi A, Schlub TE, Wheatley AK, et al. 2021. Neutralizing antibody levels are highly predictive of immune protection from symptomatic SARS-CoV-2 infection. *Nat Med*. 27(7):1205–11

4. Wang XQ, Danenberg E, Huang C-S, Egle D, Callari M, et al. 2023. Spatial predictors of immunotherapy response in triple-negative breast cancer. *Nature*. 621(7980):868–76

5. Liu D, Schilling B, Liu D, Sucker A, Livingstone E, et al. 2019. Integrative molecular and clinical modeling of clinical outcomes to PD1 blockade in patients with metastatic melanoma. *Nat Med*. 25(12):1916–27

6. Kotliarov Y, Sparks R, Martins AJ, Mulè MP, Lu Y, et al. 2020. Broad immune activation underlies shared set point signatures for vaccine responsiveness in healthy individuals and disease activity in patients with lupus. *Nat Med*. 26(4):618–29

7. Liu C, Martins AJ, Lau WW, Rachmaninoff N, Chen J, et al. 2021. Time-resolved systems immunology reveals a late juncture linked to fatal COVID-19. *Cell*. 184(7):1836-1857.e22

8. Sparks R, Lau WW, Liu C, Han KL, Vrindten KL, et al. 2023. Influenza vaccination reveals sex dimorphic imprints of prior mild COVID-19. *Nature*. 614(7949):752–61

9. Mulè MP, Martins AJ, Cheung F, Farmer R, Sellers BA, et al. 2024. Integrating population and single-cell variations in vaccine responses identifies a naturally adjuvanted human immune setpoint. *Immunity*. 57(5):1160-1176.e7

10. Gygi JP, Maguire C, Patel RK, Shinde P, Konstorum A, et al. 2024. Integrated longitudinal multiomics study identifies immune programs associated with acute COVID-19 severity and mortality. *J Clin Invest*. 134(9):

11. Klein J, Wood J, Jaycox JR, Dhodapkar RM, Lu P, et al. 2023. Distinguishing features of long COVID identified through immune profiling. *Nature*. 623(7985):139–48

12. Xiao J, Ding R, Xu X, Guan H, Feng X, et al. 2019. Comparison and development of machine learning tools in the prediction of chronic kidney disease progression. *J Transl Med*. 17(1):119

13. Mysona DP, Tran L, Bai S, dos Santos B, Ghamande S, et al. 2021. Tumor-intrinsic and -extrinsic (immune) gene signatures robustly predict overall survival and treatment response in high grade serous ovarian cancer patients. *Am J Cancer Res*. 11(1):181–99

14. Sevy AM, Soto C, Bombardi RG, Meiler J, Crowe JE. 2019. Immune repertoire fingerprinting by principal component analysis reveals shared features in subject groups with common exposures. *BMC Bioinformatics*. 20(1):629

15. Kobak D, Berens P. 2019. The art of using t-SNE for single-cell transcriptomics. *Nat Commun*. 10(1):5416

16. Becht E, McInnes L, Healy J, Dutertre C-A, Kwok IWH, et al. 2019. Dimensionality reduction for visualizing single-cell data using UMAP. *Nat Biotechnol*. 37(1):38–44

17. Sparks R, Rachmaninoff N, Lau WW, Hirsch DC, Bansal N, et al. 2024. A unified metric of human immune health. *Nat Med*

18. Wang Z, Katsaros D, Wang J, Biglio N, Hernandez BY, et al. 2023. Machine learning-based cluster analysis of immune cell subtypes and breast cancer survival. *Sci Rep*. 13(1):18962

Downloaded from www.annualreviews.org. Yale University (ar-150330) IP: 130.132.173.234 On: Tue, 07 Jul 2026 16:58:55

19. Zhang H, Yao Y, Wu J, Zhou J, Zhao C, et al. 2022. Comprehensive Analysis Identifies and Validates the Tumor Microenvironment Subtypes to Predict Anti-Tumor Therapy Efficacy in Hepatocellular Carcinoma. *Front Immunol*. 13:838374

20. Jiang Y, Xie J, Han Z, Liu W, Xi S, et al. 2018. Immunomarker Support Vector Machine Classifier for Prediction of Gastric Cancer Survival and Adjuvant Chemotherapeutic Benefit. *Clin Cancer Res*. 24(22):5574–84

21. Fourati S, Tomalin LE, Mulè MP, Chawla DG, Gerritsen B, et al. 2022. Pan-vaccine analysis reveals innate immune endotypes predictive of antibody responses to vaccination. *Nat Immunol*. 23(12):1777–87

22. Laatifi M, Douzi S, Ezzine H, Asry CE, Naya A, et al. 2023. Explanatory predictive model for COVID-19 severity risk employing machine learning, shapley addition, and LIME. *Sci Rep*. 13(1):5481

23. Kegerreis B, Catalina MD, Bachali P, Geraci NS, Labonte AC, et al. 2019. Machine learning approaches to predict lupus disease activity from gene expression data. *Sci Rep*. 9(1):9617

24. Partin A, Brettin TS, Zhu Y, Narykov O, Clyde A, et al. 2023. Deep learning methods for drug response prediction in cancer: Predominant and emerging trends. *Front Med (Lausanne)*. 10:1086097

25. Zitnik M, Agrawal M, Leskovec J. 2018. Modeling polypharmacy side effects with graph convolutional networks. *Bioinformatics*. 34(13):i457–66

26. Wei Q, Ramsey SA. 2021. Predicting chemotherapy response using a variational autoencoder approach. *BMC Bioinformatics*. 22(1):453

Downloaded from www.annualreviews.org. Yale University (ar-150330) IP: 130.132.173.234 On: Tue, 07 Jul 2026 16:58:55

27. Oh M, Zhang L. 2023. DeepGeni: deep generalized interpretable autoencoder elucidates gut microbiota for better cancer immunotherapy. *Sci Rep*. 13:4599

Downloaded from www.annualreviews.org. Yale University (ar-150330) IP: 130.132.173.234 On: Tue, 07 Jul 2026 16:58:55
